# Contemporary Management of Patent Foramen Ovale: A Multinational Survey on Cardiologists' Perspective

**DOI:** 10.1155/2021/6955791

**Published:** 2021-09-10

**Authors:** Maciej Dębski, Amr Abdelrahman, Halia Alshehri, Marloe Prince, Andrew Wiper, Shajil Chalil, Dariusz Dudek, Christopher J. White, David Hildick-Smith, David H. Roberts

**Affiliations:** ^1^Lancashire Cardiac Centre, Blackpool Teaching Hospitals NHS Foundation Trust, Blackpool, UK; ^2^Norwich Medical School and Norfolk and Norwich University Hospital, University of East Anglia, Norwich, UK; ^3^King Fahad Medical City, King Salman Heart Center, Riyadh, Saudi Arabia; ^4^Ochsner Heart and Vascular Institute, Ochsner Medical Center, New Orleans, LA, USA; ^5^Institute of Cardiology, Jagiellonian University Medical College, Krakow, Poland; ^6^Maria Cecilia Hospital, GVM Care & Research, Cotignola, Ravenna, Italy; ^7^Brighton & Sussex University Hospitals NHS Trust, Brighton, UK

## Abstract

**Objectives:**

The purpose of our survey is to analyze the clinical approach used by interventional and imaging cardiologists to diagnose, treat, and follow-up patients with PFO-related left circulation thromboembolism in different parts of the world with particular emphasis on adherence to current guidelines.

**Background:**

Firm guidelines do not cover many aspects of PFO-related patient care. Consequently, very disparate approaches exist among clinicians in the real-world.

**Methods:**

A 24-item electronic questionnaire was sent directly to experienced cardiology specialists practicing at consultant/attending positions directly involved in PFO closure management in the United States, United Kingdom, Gulf countries, and other countries. There were no unanswered questions. Responses were recorded between October 2019 and July 2020.

**Results:**

Seventy-one responses were obtained: 31 from the UK, 19 from the US, 16 from Gulf countries, 2 from Poland, and 1 response from Australia, Italy, and Switzerland. The overall response rate was 76%. Significant differences between regions were noted in the duration of ECG monitoring during the diagnostic process, PFO closure for left circulation thromboembolism other than stroke/transient ischemic attack, and intraoperative use of intracardiac echocardiography. A similar pattern was noted in the lack of routine screening for thrombophilia and the use of the long-term single antiplatelet therapy.

**Conclusions:**

The study shows a vast spectrum of opinions on the optimal approach to PFO closure with significant differences between the US, UK, and Gulf countries. The results stress the need for systematic, high-quality data on the diagnostic work-up and follow-up strategies to inform the standardized approach.

## 1. Introduction

The recent publications of long-term outcomes from randomized controlled trials and meta-analyses confirm the efficacy of PFO closure in mitigating the risk of recurrent ischemic stroke compared with the antiplatelet therapy alone in patients with cryptogenic stroke [1–5]. Based on the review of best available evidence from trials and nonrandomized data, the European Society of Cardiology (ESC) issued a position study on the management of patients with PFO and systemic thromboembolism in order to guide a rational approach to PFO management from the index event to follow-up [6]. The experts acknowledged very disparate approaches among clinicians in the real-world and stressed the need for urgent evaluation. Therefore, we aimed to capture the contemporary routine clinical practice of cardiologists specializing in PFO management and their adherence to key recommendations on an international level.

## 2. Methods

### 2.1. Study Design

A 24-item electronic questionnaire consisting of multiple choice (single or multiple answers) or open-ended questions was devised to identify practice patterns in three core areas: patient screening, procedure, and follow-up (online supplement). Senior authors identified and contacted suitable respondents in their country/region of practice. The survey was sent directly to subjects in the e-mail alongside a covering letter meeting the informed consent requirements. All respondents were experienced cardiology specialists practicing as consultant/attending physicians, and each of them was directly involved in PFO closure procedures either as the operator, imaging specialist, or in both roles. There were no unanswered questions. Responses were recorded between October 2019 and July 2020. The study was exempt from ethical approval on the grounds of informed consent, voluntary participation, and warrant of confidentiality.

### 2.2. Statistical Analysis

Data analysis was performed with SPSS, version 26.0 (IBM Corporation, Armonk, NY). Categorical variables are presented as numbers and percentages and compared using Fisher's exact test. Continuous variables are presented as median (interquartile range) and compared using the Kruskal–Wallis test. Post hoc pairwise comparisons were performed after significant effects have been found and were adjusted using the Bonferroni method. *P* < 0.05 was considered statistically significant.

## 3. Results

**Figure 1:**
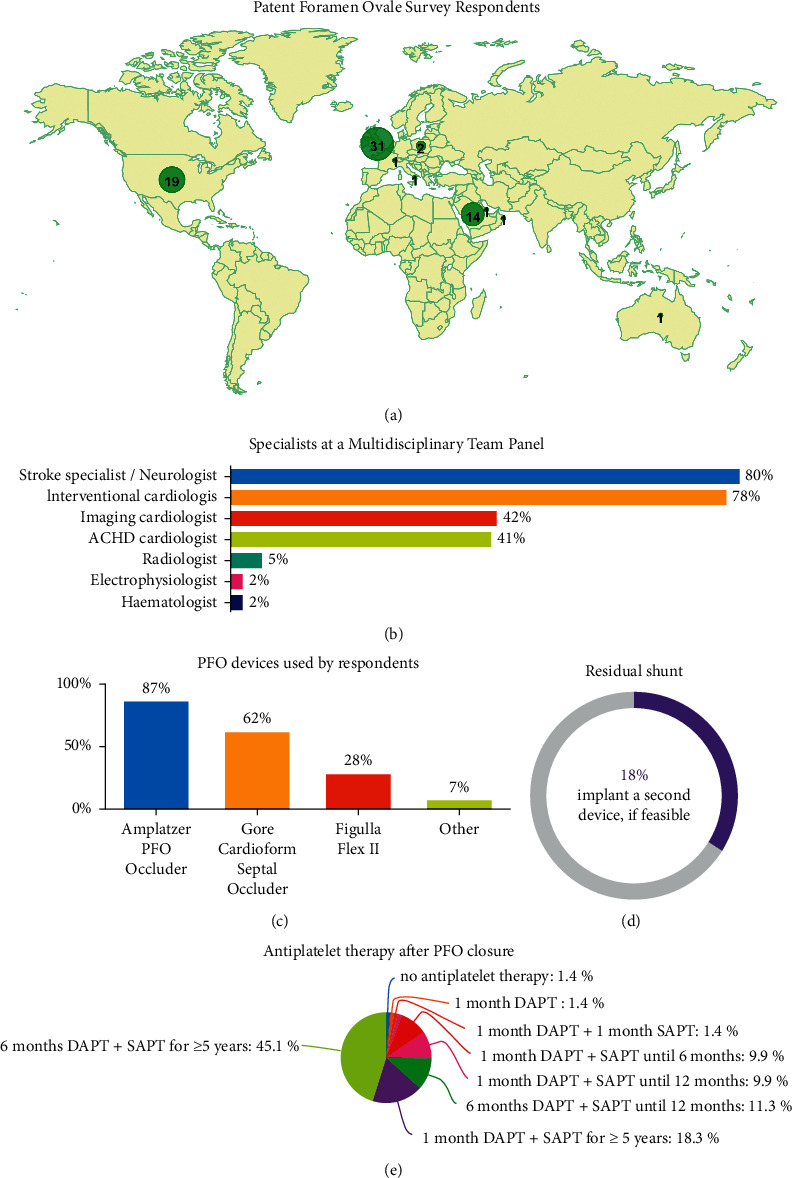
(a) Map showing the distribution of responses. (b) The presence of different specialists at multidisciplinary team meeting. (c) Patent foramen ovale devices used by the respondents. (d) Residual shunt occlusion. (e) Choice of drug therapy after patent foramen ovale closure. ACHD, adult congenital heart disease; DAPT, dual antiplatelet therapy; SAPT, single antiplatelet therapy.

Seventy-one responses were obtained from different parts of the world: 31 from the UK, 19 from the US, 16 from Gulf countries, 2 from Poland, and 1 response from Australia, Italy, and Switzerland (Figure 1(a)). The overall response rate was 76%. Regional differences between the US, UK, and Gulf countries are presented in Table 1. Experience of ten or more years in PFO closure was declared by nearly three-quarters of respondents (*n* = 52, 73%); median length of experience was 13 (7–19) years. The majority of respondents were interventional cardiologists (*n* = 55, 77%) followed by imaging specialists (*n* = 7, 10%), while 9 (13%) respondents had expertise both in PFO intervention closure and imaging. The median population served was 2.8 (1.6–5) million inhabitants, and annual operator volume was 33 (15–49) patients at the time of the survey.

### 3.1. Preprocedural Diagnostic Work-Up and Patient Selection

Silent atrial fibrillation (AF) as the cause for thromboembolism was excluded with the use of 7–30-day continuous ECG monitoring (*n* = 39, 55%), escalated to insertable cardiac monitor (ICM) if appropriate (*n* = 17, 24%). The rest of the respondents (*n* = 32, 45%) felt that short-term ECG Holter monitoring ranging from 1 to 5 days was sufficient. 6% of respondents required a routine full screen for hypercoagulable disorders before PFO closure, 27% required screening only in patients with relevant family history, and 68% declared not to screen for thrombophilia. Three-quarters of respondents performed PFO closure in patients who were above 60 years of age, and two-thirds performed PFO closure in patients who had a typical transient ischemic attack (TIA) with normal brain diffusion-weighted MRI following a positive bubble study and “high-risk” features for paradoxical embolism. PFO closure for left circulation thromboembolism to organs other than the brain was performed by 45 (63%) of whom 37 mentioned coronary arteries; 25, extremity; 16, retina; 12, mesenteric artery/intestine; 9, spleen; 2, kidney; and 1, spinal cord. The Paradoxical Embolism (RoPE) Score was used routinely by 20% and occasionally by 41%.  Figure 1(b) shows the specialists present at a multidisciplinary team (MDT) panel. The majority included interventional cardiologists and stroke physicians or neurologists but less than half included an imaging specialist. Seven respondents declared not to use an MDT process as their cases were already multidisciplinary at the point of referral.

### 3.2. Imaging and Procedural Technique

Transoesophageal echocardiogram (TOE) was used by 69% of respondents to characterize the PFO and risk stratify in the diagnostic phase. Transcranial Doppler was available for 23 (32%) of respondents. Six used it as a first-line imaging modality, 13 when the bubble study was equivocal or negative and the remainder rarely or never.

Intracardiac echocardiography and 3D imaging were used during the procedure by less than half (45% and 37%) of respondents, and the Amplatzer device was used by the majority (87%) (Figure 1(c)). The majority (61%) indicated that they deploy the device via the PFO tunnel, and if this fails, they then perform a transseptal puncture (TSP). A minority (6%) performed transseptal puncture without trying to deploy the device through the PFO tunnel, but 11% of respondents do not perform TSP and would not close very long tunnel defects percutaneously.

### 3.3. Follow-Up

Regarding approach to a residual shunt, the majority (*n* = 41, 58%) of respondents repeat bubble echocardiogram every 6–12 months until shunt closes, 26 (37%) start or continue dual antiplatelet agents, and 6 (8%) switch to anticoagulation. Thirteen (18%) cardiologists consider implantation of a second PFO device, if feasible (Figure 1(d)). Eighteen (25%) cardiologists undertake routine screening with Holter ECG monitoring for a new-onset atrial fibrillation after device implantation. The general approach to antiplatelet therapy was as follows: 97% proposed dual antiplatelet therapy (DAPT) for 1–6 months after PFO closure, and 63% continued single antiplatelet therapy (SAPT) for at least five years (Figure 1(e)). A repeat bubble echocardiogram for residual PFO was routinely performed by 27% at three months, 41% at six months, and 8% at twelve months.

### 3.4. Future Directions

PFO closure for primary prevention in patients with a very high risk of paradoxical embolization or cryptogenic ischemic stroke was declared by 11 (15%) respondents, all were non-US based. In addition, 76% of cardiologists performed PFO closure in one or more of the following indications: decompression sickness, migraine with aura, and platypnea-orthodeoxia syndrome.

## 4. Discussion

To our knowledge, it is the first survey capturing opinions of consultant cardiologists directly involved in PFO closure in different parts of the world. The previous survey on PFO closure practice included 120 physicians in the UK, the vast majority of whom were noncardiologists [7]. This survey provides an insight into current clinical practice related to selection and management in patients with left circulation thromboembolism and shows how varied cardiologists' practice is regarding PFO closure. The evidence base for PFO closure for secondary prevention in patients with left circulation thromboembolism has been elusive, and it is only recent that an official position study has been published [6]. Everything prior to this had been expert opinion rather than the fact, and this likely accounts for the considerable variation in clinical practice as observed in this survey.

Based on the responses from the UK, the US, and Gulf countries, we show that there is no standard approach to patient selection, work-up, and follow-up on the international level. In the UK, the commissioning policy for PFO closure as secondary prevention following cerebrovascular accident requires patients to be below the age of 60 and recommends that TIA be supported by imaging evidence of cerebral infarction, as per the criteria used for clinical trials [8]. Our results demonstrate that most respondents in the UK and elsewhere have performed PFO closure in patients above 60 years of age and in TIA patients without an MRI footprint. The UK Commissioning Policy also recommends that all patients will be discussed at MDT consisting of stroke specialist and interventional cardiologist with expertise in PFO management, and hereby, we show good compliance to this guidance by UK cardiologists [8]. Preferential use of at least 7-day ECG monitoring including patch-type devices and ICM to detect preexisting occult AF was declared, and this is likely to become more widespread, given the growing evidence base for extensive heart rhythm surveillance [9]. PFO closure has been associated with a four to five-fold increased risk of AF development compared with medical management [10, 11]. The latest meta-analysis showed that the magnitude of risk occurs within the first 45 days (27.2 patients per 100 patient-years) compared with the period after 45 days (1.3 patients per 100 patient-years).10 The risk of PFO device-associated stroke during this period appears to be low, however, not negligible [11]. Regarding the choice of drug therapy after PFO closure, the responses are mainly in line with ESC recommendations; almost all respondents routinely use 1–6 months of DAPT, and the majority continued SAPT for at least five years. The responses varied significantly as to whether follow-up echocardiography looking at residual shunt should be performed and what time frame is the most appropriate. A previous study shows that residual shunt is present in up to 25% of patients after PFO closure [12]. At present, there is a paucity of data on the optimal approach to residual shunt [6]. We have found a broad spectrum of approaches to residual shunt management, ranging from no action, antiplatelet agents, or anticoagulants to repeat closure with a second device. In the authors' opinion, in patients with a residual shunt, long-term SAPT should be maintained life-long, while we await further follow-up data.

Approximately one-fifth of UK and Gulf-based respondents declared having closed PFO for primary prevention of paradoxical systemic embolism, whereas none in the US. Undoubtedly, at present, there is no trial data to support this concept. On the other hand, it has been postulated that PFO closure may be beneficial in patients with high-risk anatomical PFO characteristics and a history of venous thromboembolism [13]. US and UK respondents have reported closing PFO for decompression sickness, migraine with aura, and platypnea-orthodeoxia syndrome. The circumstances where PFO closure might be considered in these conditions above have been summarised in the second part of the ESC position statement [14]. Given the weak current evidence, the multidisciplinary group of experts have urged that new prospective observational and randomized studies are needed to obtain more definitive evidence. Also, local registries providing prospective evaluations of outcomes were strongly encouraged.

## 5. Limitations

The survey insights should be interpreted cautiously as a snapshot of contemporary practice. We used purposive, nonrandom sampling to deliberately target individuals especially knowledgeable about and experienced with PFO management. While the response rate was satisfactory, the inability to identify and reach all suitable clinicians in regions of interest is a potential source of bias. Nevertheless, to our best knowledge, there had been no systematic difference in characteristics between responders and nonresponders; hence, nonresponse bias would most likely not have occurred. The specific differences in the survey results, such as length of ECG monitoring to look for preexisting occult AF, availability of ICE, and PFO closure devices, are likely due to different reimbursement models used in the three regions.

## 6. Conclusions

The study shows a very broad spectrum of opinions on the optimal approach to PFO closure. The results stress the need for systematic, high-quality data on the diagnostic work-up and follow-up strategies to inform a standardized approach. In particular, studies on the significance and optimal approach to residual shunt are warranted.

## Figures and Tables

**Table 1:** Differences between the United Kingdom, United States, and Gulf countries with respect to key aspects of PFO management (based on 66 responses).

Parameter	US (*n* = 19)	UK (*n* = 31)	Gulf (*n* = 16)	*P* value
Respondents' characteristics				
Experience (years)	18 (10–20)^*∗*^	11 (10–15)	5 (3–13)^*∗*^	0.004
Population size of a PFO service	1.5 (1–5)^*∗*^^†^	3 (2–5)^*∗*^	5 (2–23)^†^	0.002
Interventional cardiologists (PFO operators)	19 (100%)^*∗*^	31 (100%)^†^	10 (63%)^*∗*^^†^	<0.001
Annual volume	40 (20–75)^*∗*^	40 (30–50)^†^	10 (5–18)^*∗*^^†^	<0.001

Screening and patient selection				
No screening for thrombophilia	5 (26%)	6 (19%)	2 (13%)	0.616
≥7-day ECG monitoring to exclude AF including use of ICM	18 (95%)^*∗*^^†^	12 (39%)^*∗*^	7 (44%)^†^	<0.001
PFO closure in patients older than 60-year-old	18 (95%)^*∗*^	24 (77%)	7 (44%)^*∗*^	0.002
PFO closure in patients with typical TIA, high-risk features, and negative brain DW-MRI	13 (68%)	19 (61%)	9 (56%)	0.720
PFO closure of left circulation thromboembolism other than stroke/TIA	10 (53%)^*∗*^	28 (90%)^*∗*^^†^	3 (19%)^†^	<0.001
Occasional or regular use of the RoPE score	15 (79%)	14 (45%)	13 (81%)	0.013
Routine TOE before PFO closure procedure	13 (68%)	16 (52%)^*∗*^	15 (94%)^*∗*^	0.009
Availability of transcranial Doppler	9 (47%)	6 (19%)	4 (25%)	0.127

Procedure and follow-up				
Intraoperative use of ICE	18 (95%)^*∗*^^†^	11 (36%)^*∗*^	3 (19%)^†^	<0.001
Continuation of single antiplatelet therapy for at least 5 years	13 (68%)	21 (68%)	9 (63%)	0.891
Repeat bubble echocardiogram postdischarge	16 (84%)	24 (77%)	10 (56%)	0.170

Future directions				
PFO closure as a primary prevention	0 (0%)	6 (19%)	3 (19%)	0.050
PFO closure for decompression sickness/migraine with aura/platypnea-orthodeoxia syndrome	16 (84%)^*∗*^	30 (97%)^†^	5 (31%)^*∗*^^†^	<0.001

Values are median (IQR) or *n* (%). ^*∗*^ and ^†^ denote the significant difference in post hoc pairwise comparison using Bonferroni correction at adjusted *p* value < 0.05. AF, atrial fibrillation; DW-MRI, diffusion-weighted magnetic resonance imaging; ECG, electrocardiogram; ICE, intracardiac echocardiography; ICM, insertable cardiac monitor; PFO, patent foramen ovale; RoPE, Risk of Paradoxical Embolism TOE, transoesophageal echocardiography; TIA, transient ischemic attack.

## Data Availability

The survey data used to support the findings of this study are available from the corresponding author upon request.

## Supplementary Materials

Supplementary Materials A 24-item survey.
