# Supplementary material for: Contemporary Management of Patent Foramen Ovale: A Multinational Survey on Cardiologists' Perspective
Source: J Interv Cardiol. 2021 Sep 10;2021:6955791. doi: 10.1155/2021/6955791 (PMC8449721; doi:10.1155/2021/6955791)
Supplement: Supplementary Materials — A 24-item survey. [file 6955791.f1.docx]

**Supplementary material: A 24-item survey.**

- Required

**General inquiries**

- 1. **What is your role in PFO closure? ***

*Mark only one oval.*

Interventional cardiologist performing PFO closure

Cardiologist with interest in imaging assessing patients for PFO-related thromboembolism Both

Other:

- 1. **In which country do you practice? ***

*Mark only one oval.*

Australia Egypt

Gulf Countries Ireland

New Zealand Poland South Africa

United Kingdom

United States of America Other:

- 1. **How many years have you been performing/assessing for PFO closure? ***
  2. **What size of population (in millions) is your PFO closure service covering? ***
  3. **What is your centre annual PFO closure volume? ***

**Patient screening and selection**

- 1. **Screening for hypercoagulable disorder prior to PFO closure ***

*Mark only one oval.*

Patients are screened for antiphospholipid antibodies (circulating anticoagulant and anticardiolipin antibodies)

Patients who have a first degree relative with thromboembolic event prior to age 55 the following additional tests are required and must be interpreted as normal: factor V Leiden mutation, prothrombin gene G20210A mutation, protein C, protein S, antiphospholipid antibodies and antithrombin III

Patients are not required to be screened for the above Other:

- 1. **What methods are used to exclude atrial fibrillation as a cause for left circulation thromboembolism? ***

*Check all that apply.*

Holter ECG monitoring ≥ 24-hour

30-day non-invasive ECG monitoring Implantable loop recorder

Other:

- 1. **Have you referred/performed PFO closure in a patient older than 60-year-old? ***

*Mark only one oval.*

Yes

No

- 1. **Have you referred/performed PFO closure in a patient with a typical TIA, normal brain imaging including diffusion weighted brain MRI and high risk features of PFO-related thromboembolism? ***

*Mark only one oval.*

Yes

No

- 1. **Have you performed PFO closure in a patient with left circulation thromboembolism beyond the brain? ***

If 'Yes' please provide the organ/s

*Check all that apply.*

No

Coronary artery Mesenteric artery/intestine Upper extremity

Lower extremity Retina

Spleen Spinal cord

Other:

- 1. **The final decision regarding patients’ PFO closure is discussed at MDT meeting which involves? ***

*Check all that apply.*

Interventional cardiologist

Congenital heart disease cardiologist Neurologist

Stroke physician Radiologist

Cardiologist with interest in imaging Other:

- 1. **Are you using Risk of Paradoxical embolism (RoPE) scoring system to stratify patients with PFO and cryptogenic stroke by the probability that stroke was attributable to PFO? ***

*Mark only one oval.*

Yes, in each and every case Yes, occasionally

Never Other:

- 1. **Do you routinely perform TOE before PFO closure procedure? ***

*Mark only one oval.*

Yes

No

- 1. **Is transcranial Doppler (TCD) available to the referring consultants/the PFO operators? ***

*Mark only one oval.*

Yes

No *Skip to question 16.*

- 1. **If your referring consultants/you have access to TCD ultrasonography ***

*Mark only one oval.*

we use it as a first-line test for PFO detection

we use it when bubble TTE study was negative or equivocal for PFO Other:

**Procedure**

- 1. **Which device have you used for PFO closure? ***

*Check all that apply.*

Amplatzer PFO Occluder (St Jude Medical, St.Paul, MN, USA) Figulla Flex II Occlutech (Helsingborg, Sweden)

GORE® Cardioform Septal Occluder (WL Gore & Associates, Inc., Newark, DE, USA) Other:

- 1. **What imaging technique do you routinely perform during PFO closure? ***

*Check all that apply.*

2D transoesophageal echocardiography 3D transoesophageal echocardiography Intracardiac echocardiography (ICE) Other:

- 1. **In a patient with a long PFO tunnel (>12 mm) do you perform transseptal puncture through the septum primum of the fossa ovalis near the septum secundum and the tunnel origin to facilitate device deployment? ***

*Mark only one oval.*

tunnel

I am first trying to deploy the device via tunnel and if failed I perform a transseptal puncture I perform transseptal puncture without trying to deploy the device via tunnel

I have never performed transseptal puncture and generally do not close PFO with a long Other:

**Follow-up**

- 1. **What is a routine drug regimen following PFO closure? ***

*Check all that apply.*

|  | I do not use | 1 month | up to 6 months | up to 12 months | up to 5 years | up to 5 years |
| --- | --- | --- | --- | --- | --- | --- |
| I use DAPT for: |  |  |  |  |  |  |
| After DAPT I continue single antiplatelet therapy for: |  |  |  |  |  |  |

- 1. **What is your approach in case of a residual shunt following PFO closure? ***

*Check all that apply.*

I start patient on anticoagulation until shunt closes I continue DAPT until shunt closes

I repeat echocardiogram (TOE/TTE) every 6-12 months until shunt closes I implant a second device if feasible

Other:

- 1. **When is the bubble study performed following discharge providing that the procedure was successful? ***

*Mark only one oval.*

3 months

6 months

9 months

12 months

I do not repeat bubble test

- 1. **How do you screen patients for new-onset AF following PFO closure? ***

*Check all that apply.*

ECG in the hospital prior discharge

Holter ECG monitoring within 1 week of the procedure Holter ECG monitoring within one month of the procedure Other:

**Future directions**

- 1. **Do you ever perform PFO closure as a primary prevention in patients with very high risk of paradoxical embolization or cryptogenic ischaemic stroke? ***

*Mark only one oval.*

Yes

No

- 1. **Do you ever perform PFO closure as a treatment for: ***

*Check all that apply.*

Migraine with aura Decompression sickness Platypnea-orthodeoxia syndrome None of the above

Other:
